# Thought–Action Fusion in Individuals with a History of Recurrent Depression and Suicidal Depression: Findings from a Community Sample

**DOI:** 10.1007/s10608-018-9924-7

**Published:** 2018-06-04

**Authors:** B. Gjelsvik, N. Kappelmann, T. von Soest, V. Hinze, R. Baer, K. Hawton, C. Crane

**Affiliations:** 1 Department of Psychiatry, University of Oxford, Oxford, UK; 2 Department of Psychology, University of Oslo, Oslo, Norway; 3 Department of Psychiatry, Warneford Hospital, University of Oxford, Oxford, OX37JX UK; 4 University of Kentucky, Lexington, USA

**Keywords:** Thought–action fusion, Suicidal ideation, Self-harm, Suicide, Depression

## Abstract

Although suicidal ideation is one of the most consistent symptoms across recurrent episodes of depression, the mechanisms underpinning its maintenance are poorly understood. In order to develop effective treatments for suicidally depressed patients, understanding what maintains suicidal distress is critical. We hypothesised that Thought–Action Fusion (TAF), i.e., to assume that having a thought has real world consequences, originally described in Obsessive–Compulsive Disorder, might be a bias in recurrently suicidally depressed people. To assess this, we revised the original TAF scale, and assessed TAF in three samples: healthy controls, recurrently depressed individuals with no history of suicidality (D-NS) and individuals with a history of recurrent suicidal depression (D-S). Exploratory and confirmatory factor analyses indicated a three-factor solution of TAF: (1) TAF for uncontrollable events, (2) self-suicidal TAF for suicidal acts related to oneself, and (3) TAF for positive controllable events. Compared to healthy controls, the D-NS group reported significantly higher total TAF, TAF uncontrollable, and TAF self-suicidal subscales, whilst positive controllable TAF was lower compared to healthy controls. Both D-S and D-NS samples reported higher TAF for suicidal thought compared to healthy controls, i.e., believing that having suicidal thoughts means they will act on them, however in the context of low mood this became more pronounced for the D-S group. These findings suggest that targeting TAF both in suicidal and non-suicidal depression has merit.

**Electronic supplementary material:**

The online version of this article (10.1007/s10608-018-9924-7) contains supplementary material, which is available to authorized users.

## Introduction

Although suicidal cognitions are a common feature of major depression, they are also prevalent among people seeking health care (Scott et al. 2012) and observed to some degree in general population samples (Crosby et al. 2011). Interestingly, people differ in the way they experience and react to such cognitions, and whilst for some people suicidal thoughts are fleeting and temporary (Nock et al. 2009), for others they tend to persist and cause distress. Likewise, even in people with severe recurrent depression, the degree of distress that suicidal thoughts and images evoke varies markedly (e.g.; Crane et al. 2014). We have previously suggested that vulnerable individuals’ *relationship with* and *responses to* suicidal cognitions are critical in determining whether suicidal cognitions persist and potentially escalate and that such persistence is most likely when individuals respond to suicidal thoughts with rumination and suppression (Williams et al. 2016). In line with this hypothesis Pettit et al. (2009) have found that suppression of suicidal thoughts increases their severity over time.

Although it is established that differences in response to suicidal cognitions exist in patient and general population samples, relatively little is known about the factors that determine these. However, models of Obsessive–Compulsive Disorder (OCD); (e.g., Clark 1999), in which distressing and intrusive thoughts are core symptoms, may suggest potential candidate mechanisms. One relevant concept arising from the OCD literature is *Thought–Action Fusion* (TAF); (Rachman 1993; Shafran et al. 1996; Craig and Lafreniere 2016). TAF was introduced by Rachman (1993) to describe a phenomenon in which a person believes that the mere presence of intrusive thoughts can influence events in the real world. Rachman (1993) describes two components of TAF: First, ‘morality TAF’ describes the tendency to assume that the occurrence of certain intrusions implies immorality of character (e.g., intrusions about killing equal being a bad person). Second, ‘likelihood TAF’ describes the view that the mere presence of thoughts has real life consequences and could, for example, increase the likelihood of catastrophic events (e.g., intrusions about killing somebody make it more likely it will happen). TAF has been proposed to promote engagement in strategies (e.g., suppression, worry, rumination, neutralising behaviours) intended to control intrusions, prompted by the perceived probability of the thought content happening, and/or by the misinterpretation that a person is responsible for harm unless they take action to prevent it (Rachman 1993; Rassin et al. 2000). Importantly, however, since attempts to suppress unwanted thoughts have been found in many cases to lead to a paradoxical increase in their frequency (Abramowitz et al. 2001) and intensity (Wegner 1994), TAF is likely to contribute to the escalation of symptoms over time. Indeed, Rassin et al. (1999) found that experimentally induced TAF led to an increase in intrusions and perceived discomfort in healthy controls.

Whilst several studies have found a strong relationship between OCD and TAF (e.g., Amir et al. 2001; Shafran et al. 1996), some studies have shown that when depression is controlled for, the association between OCD and TAF is no longer significant (O’Leary et al. 2009; Jonsson et al. 2011). Nevertheless, to our knowledge, only two studies have examined TAF in depressed samples (Abramowitz et al. 2003; Meyer and Brown 2013). Abramowitz et al. (2003) compared TAF in OCD samples to other anxiety disorder samples, clinically depressed patients and healthy controls. They found that OCD patients had higher likelihood-TAF for others-related events compared to depressed patients, patients with social phobia and healthy controls, but not higher likelihood TAF for self-related events. However, there were no overall differences across groups on Morality TAF. Inferences about group differences across depressed and OCD samples were complicated by the OCD sample scoring higher on the Beck Depression Inventory (BDI) than the depressed sample. However, in this study depression was secondary to the focus on anxiety disorders, and the sample of depressed patients was small (n = 19). Meyer and Brown (2013), in contrast, examined TAF in a large clinical sample including both depressed and anxious patients. Using the TAF scale developed by Shafran et al. (1996), they found that both global TAF and TAF-Likelihood were more strongly associated with obsessive–compulsive symptoms than with measures of depression and worry, suggesting that to the extent that TAF is observed in depressive populations it may be present primarily where there is also thought content which has an obsessive quality.

One reason for thinking that TAF may be relevant to suicidal ideation is that it has previously been suggested that suicidal thoughts present as a form of rigid rumination (Kerkhof and van Spijker 2011; see also; Rogers and Joiner 2017), the engagement with which can be characterised as obsessive. Further, the distressing and often graphic content of suicidal urges (e.g., ‘I could hang myself in the back garden’) could be seen to have more in common with obsessions as seen in OCD (e.g., losing control and harming oneself or others), than depressed non-suicidal thoughts (e.g., ‘I’m no good’). However, the strong ruminative nature of non-suicidal depression (e.g., Liu et al. 2017) indicates that TAF might also be relevant here. Thus, there appears to be merit in exploring the potential commonalities between OCD intrusions and suicidal cognitions, and to compare this with non-suicidal depression. It is also possible that one possible reason why existing studies of TAF in depression have not yielded strong findings, is that the standard measure to assess TAF, TAF-R (Shafran et al. 1996), was developed to assess TAF in OCD, and so may be less sensitive to identify TAF in other domains, such as in depression and suicidality. Thus, it is possible that TAF may also be observed in people with depression, particularly where there are thoughts with an obsessive quality, if items were appropriately worded to elicit it.

If TAF is present in people suffering from suicidal depression what impact would this have for symptom maintenance and exacerbation? In OCD, it is suggested that TAF increases the distress associated with intrusive thoughts and compounds attempts to neutralise them (Rassin et al. 1999; Rachman 1997). However, there is no suggestion that TAF increases the actual likelihood of enactment of such thoughts. Likewise, it might be suggested that TAF related to suicidal thoughts would similarly increase distress and cognitive preoccupation, but would not increase risk of enactment. However, suicidal cognition is characterised not only by explicit thoughts and images related to suicidal acts, but also to broader cognitions including those relating to perceived burdensomeness (e.g., ‘my death is worth more than my life to others’; Van Orden et al. 2010) and entrapment (‘nobody can help me out of this mess’; Williams 2014). Although entirely hypothetical, it is possible to speculate that if TAF also operated in relationship to these thoughts, the responsibility they evoke (e.g., to unburden the family) might serve as a volitional moderator (O’Connor 2011), reinforcing and promoting suicidal distress, and facilitating the progress from thoughts to acts. To the best of our knowledge, however, no studies have systematically examined TAF in suicidal depression, nor compared TAF in this population to individuals with a history of non-suicidal depression and healthy controls.

If TAF is present in people with recurrent suicidal depression, a second issue concerns its mood dependence. Cognitive science accounts of recurrent depression (i.e., the Differential Activation framework) posit that in people with a history of recurrent depression, depressive and suicidal thoughts can be reactivated by subtle and transient changes in mood (Williams et al. 2016; Scherrer et al. 2014; Brockmeyer et al. 2012). Indeed, mood-dependent changes in dysfunctional thinking have been shown to predict depressive relapse in previously depressed patients (Segal et al. 2006). Such reactivation involves not just negative *content* but also maladaptive *cognitive processes*. For example, our previous work has shown that people with a history of suicidal depression can be distinguished from those with a history of non-suicidal depression in the extent to which increases in negative mood impair interpersonal problem solving, a cognitive deficit characteristic of people at times of suicidal crisis (Williams et al. 2005). Likewise, people with a history of suicidality report more hopeless thoughts when asked to imagine being in a slightly sad mood, and this measure of hopelessness reactivity predicts actual cognitive deficits in positive future thinking after an experimental mood challenge (e.g.; Williams et al. 2008). Some forms of mood-dependent dysfunctional thinking (e.g., entrapment) have also been shown to increase the risk of suicidal behaviour longitudinally (O’Connor et al. 2013). If TAF is also observed in people with a history of recurrent suicidal depression, a first step in examining whether this bias predicts subsequent suicidal relapse is to investigate whether such potential endorsement of thoughts increases in the context of transient low mood. Identifying factors uniquely affected by changes in mood is helpful in understanding what serves to maintain and progress suicidal crises, and in formulating potential clinical targets. Indeed, although as stated above there is relatively little research exploring TAF in depression, Shafran et al. (1996) suggest a reciprocal relationship between mood disorders and TAF, in which depression might increase the occurrence and believability of thoughts, and in which TAF, in turn, might exacerbate low self-esteem, depression and anxiety.

Thus, there appears to be a strong rationale for exploring the potential presence of TAF in individuals with a history of suicidal depression, including the extent to which TAF might be mood-state dependent, and elevated at times of low mood or crisis. However, since the original TAF scale was developed for OCD, for the present study we considered that a revision of the items would be required in order to make the scale more amenable to depression and suicidality.

### Assessment of Thought–Action Fusion

The first version of the Thought Action Fusion Scale (Shafran et al. 1996) was explicitly developed for use with OCD patients, and consisted of 34 items, which, following factor analyses and revisions, led to the 19 item Thought–action fusion scale-revised (TAF-R). This scale consisted of three subscales all denoting negative events: TAF-moral (12 items), TAF-likelihood-self (i.e., TAF likelihood for self-related events; 3 items) and TAF-likelihood-other (i.e., TAF likelihood for self-related events: 4 items). Overall, the TAF literature shows that the three factors of TAF-R have good reliability, as reflected in Cronbach’s alphas ranging from 0.75 to 0.89 (Rassin et al. 2001).

Whilst it seems clear that patients suffering from anxiety disorders report higher TAF than healthy controls on the probability dimension (e.g., Rassin et al. 2001), comparisons with other clinical groups are sparse. Two revisions of the original scale have since been made; one including TAF for thoughts about *positive* events happening to self and others, across domains of both gain and harm avoidance (Craig and Lafreniere 2016), and one including items about likelihood of preventing harm by means of positive thoughts (Amir et al. 2001). To our knowledge, no TAF scale has been developed to specifically assess TAF for depression or suicidality.

Shafran et al. (1996) have noted that responses on the TAF tended to be idiosyncratic, and that the scale would be most usefully considered as a clinical tool to identify idiosyncratic beliefs, rather than generating sum-scores. Moreover, they emphasised that the construct of TAF was in its early stages and that associated metacognitions, specificity to OCD, and clinical implications were still unclear. Furthermore, with the notable exception of Craig and Lafreniere’s (2016) examination on TAF for positive events, the TAF literature has focussed solely on negative events (e.g., being in a car accident, either self or other; Shafran et al. 1996). Another important dimension which has not been assessed in existing versions of the TAF-R but which might influence the extent to which TAF is observed is the controllability of the event to which a thought relates. There is some evidence that thinking about an event increases the likelihood of engaging in that behaviour at least for desirable actions (e.g., imagining to vote and subsequently doing so; Libby et al. 2007), and thus there is to some degree a reality to the belief that thinking about a potentially self-initiated action may increase its probability. In contrast, there is no evidence that thinking about uncontrollable actions has any bearing on one’s behaviour. As such it is possible that pathological TAF may be most easily observed when it relates to the likelihood of other-initiated actions (e.g., friend committing a crime) or self-relevant uncontrollable actions (e.g., being called to Jury duty) for which there is no logical mechanism through which thought might lead to action. Controllability may be a particularly relevant aspect of TAF in the context of both suicidal and non-suicidal depression given the emphasis on locus of control in depressive and suicidal symptomatology. Thus, both the self-other and controllable-uncontrollable dimensions appear important in understanding the phenomenon in the context of recurrent suicidal and non-suicidal depression. Finally, in terms of convergent validity, despite the theoretical emphasis on TAF eliciting control strategies, little is known about relationship of TAF to relevant cognitive strategies other than obsessionality, such as suppression, rumination as well as a divergent construct, trait mindfulness (i.e., the capacity to pay attention to momentary experience with acceptance and non-judgement, Baer et al. 2006, 2008).

The objectives of the present study were therefore to examine TAF in suicidal and non-suicidal depression as compared to healthy controls, and also to examine the effect of a mood induction within the two depressed groups. To address the above issues concerning the measurement of TAF we (a) developed and validated a TAF scale adapted to include items whose content was relevant to suicidal depression (including items with both positive and negative content, including suicide related content), and which included items covering both self-other and controllable-uncontrollable dimensions using exploratory and confirmatory factor analysis, (b) compared the derived TAF scale in individuals with a history of suicidal depression and non-suicidal depressed compared to non-clinical controls, and (c) examined whether TAF increased in the depressed groups when these groups were asked to respond from the perspective of a time of crisis, as compared to their baseline responses. We hypothesised (1) that total TAF score would correlate positively with suppression and rumination and correlate negatively with facets of trait mindfulness across the sample as a whole, (2) that total TAF would be related linearly to lifetime depression severity (i.e., increased in depressed controls compared to healthy controls and highest in individuals with a history of suicidal depression), (3) that if a factor emerged that reflected item content related to suicidality that this factor would be higher in individuals with a history of suicidal depression than in non-suicidal depressed and healthy controls and compared to other factors for this group due to its salience, and (4) that TAF would increase for clinical groups when reported in the context of low mood as compared to baseline.

## Methods

### Participants

The current study reports on a cross-sectional online survey of adults. All participants were recruited online. Participants were recruited from the following three groups: Individuals who were currently well but had a history of suicidal depression (D-S) or non-suicidal depression (D-NS); and Healthy Controls (HC). Participants were eligible for inclusion if they were (a) between 18 and 70 years of age, (b) fluent in English, (c) currently well at the time of inclusion i.e., meeting the National Institute on Mental Health guidelines for recovery or remission (Frank et al. 1991, including minimal symptoms of depression at no more than a mild level for the previous 8 weeks) and (d) able to provide informed consent. For both the D-S and D-NS groups, the following additional inclusion criteria applied: A minimum of ≥ 3 episodes of depression, defined by DSM-IV criteria for a history of Recurrent Major Depression, of which two must have occurred within the last 5 years and at least one within the last 2 years, and being currently well defined as meeting the National Institute on Mental Health guidelines for recovery or remission (Frank et al. 1991) at the time of inclusion (i.e., minimal symptoms of depression at no more than a mild level for the previous 8 weeks). Inclusion in the D-S group required a reported history of recurrent suicidal ideation and/or behaviour. Participants were excluded if they reported current or past symptoms of (a) obsessive–compulsive disorder (OCD), (b) substance abuse, (c) bipolar disorder (including manic episodes during the last 6 months), and (d) a diagnosis of schizophrenia.

### Procedure

Participants were recruited through adverts in newsletters and flyers in the community and online, calling for healthy controls as well as individuals with a history of suicidal and non-suicidal depression but who were currently well (see above). The survey link was active from March through August 2016. Prior to participation, individuals self-screened online to ascertain that they were all currently well, and to ensure appropriate assignment to one of the following three groups: Depressed-suicidal (D-S); individuals who were currently well but had a history of suicidal depression, Depressed-Non-Suicidal (D-NS); individuals with a history of depression but no suicidality, and Healthy Controls (HC).

We recruited participants who were currently well or in remission (as above) at entry to the study to enable us to explore both (a) whether there are trait differences (either pre-existing first onset of depression or persisting as a scar effect) between individuals with a history of depression and those without and (b) whether there are differences between participants’ responses when they reported on TAF at baseline and when asked to imagine a time of crisis. Moreover, assessing TAF for suicidal events in close proximity to episode of self-harm would have been confounded with the event in question just having taken place. Assessing individuals in remission from depression minimised this risk. The protocol for this study was reviewed and approved by Oxford University’s Central University Research Ethics Committee (CUREC; protocol number MSD-IDREC-C2-2014-030).

Following online screening, eligible participants were informed about the study procedures through an electronic information sheet, and consent was obtained electronically before proceeding to complete the survey. Upon completion participants received a £10 Amazon voucher via email as reimbursement.

### Measures

#### Thought–Action Fusion-Suicidal Revision (TAF-SR)

The first version of the Thought Action Fusion Scale (Shafran et al. 1996) was explicitly developed for use with OCD patients, and consisted of 34 items, which, following factor analyses and revisions, led to the 19 item TAF-R. TAF-Suicidal Revision was based on this 19-item TAF-R (Shafran et al. 1996). As Morality TAF is not directly related to the occurrence of actual events in the real world but rather reflects a belief about the significance of events, we focussed exclusively on the likelihood aspect of TAF due to our interest in measuring people’s beliefs about their thoughts having a direct influence on outcomes in the real world. Moreover, we were interested in whether TAF for suicidal content was different from TAF for other content; thus, a revision was required. We expanded the original self-other dimension (i.e., events happening to oneself or to friends or relatives) with (a) valence of events (i.e., positive versus negative) (b) whether events were suicidal or non-suicidal and (c) controllability of events (e.g., eating healthily versus plane crash). In order to validate these additional parameters, participants rated the degree to which they found the item phrasing to reflect controllability (i.e., the degree to which the event was extraneous or self-determined) and valence (negative or positive), respectively, on a VAS scale (i.e., from 0 to 100) (see Table S2). Items were mirrored; i.e., there was an equal number of items describing events related to self and other, respectively. The original revision consisted of 30 items rated on a 5-point Likert scale from 1 (*Disagree strongly*) to 5 (*Agree strongly*), thus resulting in a possible score range from 30 to 150. The items are outlined in Table 1. To capture potential mood-dependency of TAF in the clinical groups, we applied the same principle as other questionnaires assessing vulnerability in the context of low mood (e.g. the Anxiety Sensitivity Index; Peterson and Reiss 1992; the Leiden Index of Depression Sensitivity; Van der Does 2002), assessed independently of an experimental context, asking conditional questions about their worst ever crisis in addition to questions about their current state. Thus, participants completed the same scale twice: Participants in the D-S and D-NS group (but not healthy controls) were asked to first complete the TAF scale according to how they were currently feeling (e.g., ‘How well do the following statements describe how you *are currently feeling*?’), and subsequently to complete them from the viewpoint of their worst (depressive and/or suicidal) crisis (i.e., ‘Now we would like you to describe how well the statements describe how you typically feel *at a time of crisis*’). This was based on the assumption, derived from previous work, that taking the perspective of a previous crisis would enable individuals to respond in a way that mirrors responses occurring after experimentally induced mood (e.g. Williams et al. 2008), expected to affect their responses on the revised TAF scale.

**Table 1:** Thought action fusion items, factor loadings and item-total correlation for second random half of the sample (n = 181)

No.	Description		Factor loadings			Factor^†^	Item-total correlation
	“If I think [ ],	this increases the chance [ ].”	Factor 1	Factor 2	Factor 3		
1	of myself having fun on a holiday	that I will have fun on a holiday	− 0.11	− 0.12	0.73	PC	0.16
2	of myself donating to charity	that I will donate to charity	− 0.11		0.76	PC	0.27
3	of myself eating healthily	that I will eat healthily	− 0.15		0.62	PC	0.14
4*	of myself lying to someone	that I will lie to someone	0.15	0.32	0.12	–	0.47
5*	of myself cheating on my taxes	that I will cheat on my taxes÷	0.53		0.11	–	0.63
6*	of myself deliberately parking illegally in a disabled car parking space	that I will deliberately park illegally in a disabled car	0.51			–	0.58
7	of myself winning the lottery	that I win the lottery	0.69			UC	0.74
8*	of a stranger doing something kind for me	that this will happen	0.48		0.22	–	0.58
9	of myself finding money on the street	that I will find money on the street	0.68			UC	0.72
10*	of myself becoming ill	that I will become ill		0.18	0.32	–	0.38
11	of my house being burgled	that my house will be burgled	0.73	0.11		UC	0.83
12	of myself being in a plane crash	that I will be in a plane crash	0.78			UC	0.75
13	about me killing myself	that I will kill myself		1.04		SS	0.60
14	about harming myself	that I will harm myself		0.60		SS	0.43
15	about harming myself with the intention to die	that I will harm myself with the intention to die	0.16	0.76		SS	0.65
16	of a relative/friend having fun on a holiday	of him/her having fun on a holiday	0.80	− 0.12		UC	0.73
17	of a relative/friend donating to a charity	of him/her donating to a charity	0.71			UC	0.70
18	of a relative/friend eating healthily	of him/her eating healthily	0.78			UC	0.72
19*	a relative/friend lying to someone	that he/she will lie to someone	0.86			–	0.83
20*	of a relative/friend cheating on their taxes	that he/she will cheat on their taxes	0.91			–	0.84
21*	of a relative/friend deliberately parking illegally in a disabled car parking space	that he/she will deliberately park illegally in a disabled car parking space	0.82		− 0.14	–	0.76
22	of a relative/friend winning the lottery	he/she will win the lottery	0.90			UC	0.84
23*	of a stranger doing something kind for a relative/friend	that this will happen	0.83			–	0.78
24	a relative/friend finding money on the street	that he/she will find money on the street	0.87	− 0.10		UC	0.79
25*	of a relative/friend becoming ill	that he/she will become ill	0.82		− 0.16	–	0.79
26	of a relative/friend’s house being burgled	that his/her house will be burgled	0.76			UC	0.79
27	of a relative/friend being in a plane crash	that he/she will be in a plane crash	0.75			UC	0.78
28	about a relative/friend killing themselves	that he/she will kill themselves	0.82			UC	0.86
29	about a relative/friend harming themselves	that he/she will harm themselves	0.88			UC	0.83
30	about a relative/friend harming themselves with the intention to die	that he/she will harm themselves with the intention to die	0.88			UC	0.86

*These items were removed from the scale after exploratory factor analysis

^†^Extracted factors correspond to uncontrollable (UC), self-suicidal (SS), and positive controllable (PC)

#### The Ruminative Response Scale

The Ruminative Response Scale (RRS, Nolen-Hoeksema and Morrow 1991) is a 22-item self-report scale assessing different facets of self-reflective thinking. Participants are asked to rate how much they engage in different cognitive responses on a 4-point scale, ranging from 1 (*never*) to 4 (*always*). Treynor, Gonzalez and Nolen-Hoeksema (2003) identified three subscales of the RRS: *depression-related rumination* (12 items, e.g., “think about how hard it is to concentrate”), *brooding* (five items, e.g., “think ‘Why can’t I handle things better?’”), and *reflection* (five items, e.g., “Analyze recent events to try and understand why you are depressed”). The RRS shows acceptable internal consistency for brooding (α = 0.68), and good internal consistency for depression-related rumination (α = 0.86) and reflection (α = 0.73) (Brennan et al. 2015).

#### The Five-Facet Mindfulness Questionnaire

The Five-Facet Mindfulness Questionnaire (FFMQ; Baer et al. 2006, 2008) consists of five facets assumed to be key aspects of mindfulness as a dispositional variable (Observing, Describing, Non-Judging of inner experience, Non-Reactivity to inner experience and Acting with awareness). The five facets are measured by rating how true each item within each facet is for participants on a general basis on a 5-point scale ranging from 1 (*never or very rarely true*) to 5 (*very often or always true*). The internal consistency for the scale overall has been shown to be good, with Cronbach’s alphas ranging from 0.75 to 0.91 (Baer et al. 2006), and the five facets have shown good to excellent internal consistency (Cronbach’s alphas ranging from 0.75 to 0.91). However, a four-factor model has been shown to have a better fit in non-meditating samples (e.g., Siegling and Petrides 2016) and we therefore performed subscale analyses.

#### The White Bear Suppression Inventory

The White Bear Suppression Inventory (WBSI; Wegner and Zanakos 1994) is a 15-item self-report inventory which assesses chronic thought suppression using a 5-point scale (1 = “strongly disagree” to 5 = “strongly agree”). Psychometric properties show good internal consistency (α = 0.89) (Muris et al. 1996). Factor analyses indicate that the 15-item scale consists of two related factors: the tendency to suppress thoughts, and the frequency of experiencing unwanted intrusive thoughts (e.g., Schmidt et al. 2009), indicating that a high frequency of intrusions does not necessarily equal a high level of suppression. We used the suicidal revision developed by Pettit et al. (2009) for the D-S group, and the original scale for the healthy controls and D-NS group.

### Data Analysis

#### Exploratory Factor Analysis

All data analyses for the exploratory factor analysis (EFA) were performed by using the *psych* package (Revelle 2014) in R 3.4.1 (R Core Team 2017). First, principal axis factor analysis in conjunction with parallel analysis was run using maximum likelihood estimation to determine the number of factors for factor analysis. As recommended, we used parallel analysis in conjunction with a scree plot and Kaiser’s criteria (eigenvalue > 1 rule) to have multiple criteria for determining the number of factors (Hayton et al. 2004). Results from principal axis factor analysis informed EFA that was conducted on the determined number of factors. The sample was split randomly into two subsamples. EFA was then performed on one subsample, again applying maximum likelihood estimation and promax rotation to allow for correlation between factors. Based on EFA results, items were explored on uniqueness and factor loadings to identify items that could be deleted.

#### Confirmatory Factor Analyses

To validate the solution identified by EFA, confirmatory factor analyses (CFA) were performed on the second half of the sample using MPlus 7.4 (Muthén and Muthén 2007–2014). Model fit was evaluated by inspecting χ^2^ statistics, the comparative fit index (CFI), the Tucker–Lewis index (TLI), and the root mean square error of approximation (RMSEA). According to recommendations in the literature, CFI and TLI values of 0.95 or greater and RMSEA values of 0.06 or lower were considered as indicating good fit (Hu and Bentler 1999). Robust maximum likelihood estimation procedures were employed to account for non-normality.

#### Internal Consistency and Convergent Validity

To assess internal consistency, we computed Cronbach’s α and Omega Total for the total score and factor-based subscales. Omega Total was chosen as additional metric for internal consistency since assumptions for Cronbach’s α are rarely met in practice (McNeish 2017). Convergent validity was assessed by computing Pearson correlations of TAF total and factor-based subscales with other scales. Correlations were compared using Fisher’s z-transformation.

#### Group Differences in TAF

To analyse differences in TAF between groups, we performed multiple linear regression analyses on TAF scores as dependent variable and group membership as independent variable. Analyses were conducted both unadjusted and adjusted for potential confounding variables (i.e., age and gender). Instead of regression coefficient p-values based on the t-distribution, p-values were computed by means of permutation analysis using the *lmPerm* package in R (Wheeler et al. 2016), which avoids relying on (often unmet) assumptions of homogeneity of variances and normality (Kabacoff 2015) and is similar to bootstrapping analyses.

#### Comparison of TAF in Normal and Crisis State

To examine whether there was a significant difference in the degree to which the two clinical groups’ TAF scores changed as a result of the mood induction, linear regression analyses were conducted with the TAF scores in the mood induction condition as outcome variable, with predictors being group membership (0 = D-NS, 1 = D-S), the TAF scores before mood induction, and the interaction term of theses variables. Again, p-values were estimated using permutation-based regression.

## Results

### Participant Characteristics

The sample consisted of *N* = 361 individuals, consisting of 130 HC, 134 D-NS, and 97 D-S individuals (the random subsamples consisted of *N* = 181 and *N* = 180, respectively). Baseline characteristics of the full sample and subgroups are shown in Table S1. Gender, education, ethnicity, and employment differed significantly across subsamples, with significantly higher number of men in the depressed non-suicidal group (*p* < 0.05).

### Factor Analysis

The number of factors obtained by principal axis factor analysis was cross-validated using the Kaiser criteria (3 factors > eigenvalue of 1), a scree plot (3 factors above break), and parallel analysis (4 factors > 95th percentile of simulated eigenvalues; see Fig. S1). Although parallel analysis diverted from a 3-factor solution, this solution was taken forward since the difference between observed and simulated eigenvalues for the fourth factor was negligible. EFA of this 3-factor solution with maximum likelihood estimation and promax rotation revealed a relatively clear cut factor structure (see Table 1): Factor 1 (Uncontrollable TAF) combined 24 items describing uncontrollable events related to either self and/or other (e.g., being in a plane crash, winning the lottery, others’ self-harm), Factor 2 (Suicidal TAF) combined three items that were related to own self-harm or suicide (i.e., not others’ self-harm) and were hence controllable (logically, if not phenomenologically), and Factor 3 (Controllable TAF) combined three items with self-related positive content that were also controllable (e.g., myself having fun on a holiday). The percentage of variance explained by these factors was 44.1, 7.5, and 5.8 for factors 1, 2, and 3, respectively.

The number of items was reduced from 30 to 20 items based on low factor loadings (5 items below 0.5) as well as keeping with item mirroring in the self-other category (5 corresponding items; see Table 1). All item reductions were from the uncontrollable TAF subscale.

Next, CFA was conducted on the reduced 20-item version of the TAF-SR. More specifically, a model with three correlated latent factors, where all 14 items describing uncontrollable events loaded on one factor, the three items related to self-harm loaded on another factor, and the three items describing controllable positive events loaded on a third factor. Results indicated good model fit; χ^2^(167) = 250.52, CFI = 0.95, TLI = 0.95, RMSEA = 0.053 (90% CI 0.039–0.066) of the three-factor solution. We compared the three-factor model to a single factor model where all items loaded on one overall TAF factor. However, fit for this model was not adequate; χ^2^(170) = 429.18, CFI = 0.85, TLI = 0.83, RMSEA = 0.092 (90% CI 0.081–0.103).

### Convergent and Discriminant Validity

Cronbach’s α and Omega Total showed good to excellent internal validity for total TAF (α = 0.94, ω = 0.94), uncontrollable TAF (α = 0.96, ω = 0.96), and self-suicidal TAF (α = 0.86, ω = 0.87). Internal consistency was only moderate for positive controllable TAF (α = 0.68, ω = 0.69).

Associations between TAF and other scales are presented in a correlation matrix in Table S3. Total TAF score, uncontrollable TAF, and self-suicidal TAF correlated moderately with RRS. Looking at RRS subscales (Table S4) revealed somewhat stronger correlations between TAF scales and the Reflection and Depression subscales as compared to the Brooding subscale (z = 2.84,* p* < 0.01). Correlations to other scales (BRFL, WBSI, SMQ, FFMQ) was low. However, subscale analyses for FFMQ and WBSI yielded somewhat different findings. There was a significant moderate correlation between the Describing subscale of the FFMQ and TAF total (r = .4) and TAF self-suicidal (r = .32) (Table S4). The correlation matrix (Table S4) indicates that TAF was moderately correlated with WBSI: Unwanted Intrusive Thoughts (TAF total: r = .38, TAF Uncontrollable: r = .4) but not with the other WBSI subscales Thought Suppression (r = − .05) and Self-Distraction (r = − .08). Moreover, WBSI-Unwanted Intrusive Thoughts was moderately correlated with TAF self-suicidal (r = .31).

### TAF Across Groups

Results from multiple regression analyses in conjunction with permutation tests revealed significant differences in TAF between groups (see Table S5). The D-NS group had significantly higher total TAF, uncontrollable TAF, self-suicidal TAF, and lower positive controllable TAF when compared with healthy controls. Contrary to our hypothesis, the D-S group did not show differences to HCs on overall TAF, uncontrollable TAF, and positive controllable TAF, but did show significantly higher self-suicidal TAF.

### Differences Between Normal and Crisis State

Finally, we tested whether the two clinical groups differed in how their TAF scores changed from normal to crisis states using permutation-based linear regression of outcome crisis state TAF with predictors being dummy-coded group, normal state TAF, and their interaction. D-NS individuals increased significantly more than D-S individuals from normal to crisis states on total and uncontrollable TAF scores. In contrast, the D-S group increased significantly more for suicidal TAF and decreased significantly more for the TAF positive controllable (Table S6).

## Discussion

Twenty years ago, Shafran et al. (1996) suggested that TAF might reinforce depressive symptoms in mood disorders; however, to our knowledge, only two studies have since examined TAF in depression (Abramowitz et al. 2001; Meyer and Brown 2013). The goal of this study was to compare TAF in people with a history of suicidal depression to TAF in individuals with a history of non-suicidal depression and healthy controls, and to revise and adapt the Thought–Action Fusion Scale (Shafran et al. 1996) for this purpose. To our knowledge, this is the first study to demonstrate thought-action fusion in both non-suicidal and suicidal depression. Our study produced three major findings, described below.

### 1. Development of Thought–Action Fusion Scale-Suicidal Revision

The first set of findings relates to adaptation of the original TAF scale to suicidal and non-suicidal depression. The factor analysis pointed to controllability of events as a key parameter for TAF in this sample. This is reflected both in Uncontrollable and Controllable TAF, and TAF for suicidal events, which are logically controllable, yet not necessarily perceived as such given the typically decreased sense of agency in this population. These findings extend the existing TAF literature, which has focussed solely on uncontrollable events (e.g., car accident, being injured in a fall, falling ill).

Test of convergent and discriminant validity indicated that TAF was not associated with total WBSI score, contrary to our hypothesis. However, TAF was positively correlated with the WBSI subscale ‘unwanted intrusive thoughts’, in keeping with Rassin et al.’s (1999) suggestion that TAF is linked to the tendency to experience a high frequency of intrusions, and empirical evidence that TAF predicts frequency and perceived controllability of unwanted mental intrusions (Purdon and Clark 1994). This introduces the possibility that TAF is related not just to perceived controllability of an imagined event (e.g.; perceiving of being in a plane crash as uncontrollable), but also to the perceived controllability of the cognition itself (e.g., I can’t stop my thoughts). In addition to associations with unwanted intrusions, TAF (both total and subscales) was strongly positively correlated with rumination. These findings suggest acceptable convergent validity, and lends partial support to Rachman’s (1997) hypothesis of a link between TAF and rumination. However, it is not possible to draw conclusions about the potentially causal nature of the association on the basis of this dataset. Further research is required to better understand the mechanisms linking TAF to rumination and perceived intrusiveness of thoughts, and the direction of causality.

The correlation between the *reflection* subtype of rumination and TAF was significantly stronger than between brooding and TAF. Brooding has been shown to have a stronger association with recurrent depression than reflection and to be linked to poorer outcome longitudinally (Treynor et al. 2003). However, both reflection and TAF might lock thoughts at an abstract level of cognition, thereby reinforcing an abstract-analytical style of processing and potentially reinforcing existing deficits in problem-solving-indeed, reflection has been found to be linked to suicidal ideation in patients with a history of suicide attempts (Surrence et al. 2009).

Given the poor fit of a five-facet model to non-meditator samples (e.g., Curtiss and Klemanski 2014), subscale analyses of FFMQ were considered more relevant in this context. The negative correlation between TAF total and the FFMQ subscales Describing and Acting with awareness partially corroborates the discriminant validity of the scale, given the emphasis of these facets on de-identification with thought.

The excellent model fit for the hierarchical CFA indicates that the total TAF factor represents the three factor-based subscales well. However, the low factor loading with Positive Controllable TAF suggests that, whilst from a statistical point of view the overarching TAF score is valid, in practical terms the Positive Controllable TAF items contribute only to a minor degree to the overall TAF factor. Unlike Craig and Lafreniere’s scale, there was no evidence of a distinct positive TAF. However, in their work on Positive TAF, Craig and Lafreniere (2016) differentiated between positive-gain and harm-avoidance categories, whereas in this study we focussed on positive-gain only. Given the large proportion of clinical subjects, including harm-avoidance might have yielded different results.

### 2. Differences in TAF Between Groups

Concerning the second main finding, we found important differences in Total TAF between clinical groups and healthy controls. However, our hypothesis that TAF would be highest in D-S, intermediate in D-NS and lowest in HC was not confirmed. Indeed, whilst total TAF was relatively similar in D-S and healthy controls, total TAF and Uncontrollable TAF were significantly higher in the D-NS group. Whilst problem-solving and goal-directed behaviour are likely to be impaired in individuals with a history of recurrent depression, the D-NS appear to have an elevated belief in their capacity to control the world through their thinking. Such a belief may parallel with the positive beliefs about rumination and problem-solving as regulatory behaviour (e.g., Watkins and Baracaia 2001) but appears to extend to the relationship between participants’ thoughts and the occurrence of events outside their own and other’s control. The D-S group, on the other hand, does not show this bias. One possibility is that for those with recurrent depression, general TAF is actually protective, and that in its absence, repeated negative experiences will result in the high levels of hopelessness characteristic of those who experience suicidal ideation or engage in suicidal behaviour.

Contrary to our hypothesis, there was also no basis in the findings for distinguishing between the D-S and D-NS group on Self-Suicidal TAF when participants completed the measure reporting from a perspective of being well. It is possible that Self-Suicidal TAF is underpinned by different beliefs in these two groups, e.g., that D-NS perceive them as more controllable than the D-S group, however, this awaits further scrutiny. Moreover, the prediction that for the D-S group, the salience of suicidal thoughts would override the self-other distinction was not met, indicating that it is primarily in thinking about self-relevant, suicidal themes that TAF is activated.

### 3. TAF Before and After Mood Induction

Our findings show that TAF increases in both clinical groups following a mood induction. The mean changes across states and groups (Table S6) suggest possible mood-dependency of self-suicidal TAF. The increase in Total and Uncontrollable TAF from normal to crisis states in the D-NS group (compared to the D-S group) suggests that as D-NS become distressed they increase their belief in their capacity to control events and occurrences through their thoughts. In contrast, self-suicidal TAF *increased* and Positive Controllable TAF *decreased* from normal to crisis states in the D-S relative to the D-NS group, suggesting that as the D-S group become more distressed they experience not only more suicide-related TAF but a reduction in their belief in their capacity to influence positive controllable events through their thoughts. Diminished positive controllable TAF may reflect a rise in hopelessness occurring when in the imagined crisis state, in keeping with a differential activation theory of hopelessness/suicidality (Williams et al. 2008). Together with findings of group differences, these data suggest that that differences in TAF between D-NS and D-S individuals become more pronounced in the context of low mood. This points to suicidal and positive controllable TAF as useful clinical targets.

### Limitations

The current findings need to be interpreted in the context of the following limitations: First, the study is limited by sample size (*N* = 361). Common rule of thumbs for CFA include, but are not limited to:* N* ≥ 200, and ratio of* N* to the number of variables (*p*),* N*/*p* ≥ 10 (Myers et al. 2011). In the CFA, there were 167 free parameters, whereas the convention is five participants per free parameter (Bentler and Chou 1987). However, such rules of thumbs have been widely criticised as having limited validity when applied to real data, as adequate sample size for both EFA and CFA rely on factors that would typically vary across studies (Myers et al. 2011). Replications in larger samples are required. Second, the study was limited by its cross-sectional design, and we cannot establish causality between TAF and suicidal and non-suicidally depressive distress. However, we have introduced a theoretical rationale for why TAF might be critical in the suicidal trajectory, and the results suggest that this might also apply to maintenance of non-suicidal depression. This awaits scrutiny in studies adopting experimental and longitudinal designs.

Third, given that the survey took place entirely online, the mood induction was carried out outside an experimental context. Thus, we cannot ascertain that the conditional questions entailing revisiting a time of crisis genuinely led to a drop in mood or simply encouraged participants to respond ‘as-if’ in such a mood. Findings relating to changes from baseline to crisis state requires experimental validation. Nevertheless, questionnaires adopting conditional questions (e.g., the LEIDS; Van der Does 2002) have been able to reliably distinguish between individuals with and without a history of depression, thereby suggesting that mere questions outside the experimental context have the power to induce transient dysphoric mood or enable individuals to respond in a way that mirrors responses occurring after experimentally induced mood (e.g.; Williams et al. 2008). Future studies are required using larger clinical and non-clinical samples and experimental designs in which predictions about mood dependency of TAF and its impact on other key maintenance mechanisms can be tested. Fourth, the current data set did not enable differentiating TAF in those with a history of recurrent suicidal ideation from those with a history of suicidal behaviour. Potential differences await scrutiny.

## Conclusion

It is possible that TAF reinforces an abstract-analytical style of processing involved in the maintenance of depressive (Watkins 2008) and suicidal distress (Williams et al. 2016). If so, TAF would be an obvious clinical target. However, this awaits further experimental and longitudinal scrutiny. Studies exploring TAF modification are scarce (see Jonsson et al. 2011; Siwiec et al. 2017), and in furthering our understanding of the feasibility of modifying TAF in depressed and suicidal populations, the link between TAF, problem-solving, executive functioning and cognitive flexibility require further experimental scrutiny.

## Electronic supplementary material

Below is the link to the electronic supplementary material.

Supplementary material 1 (DOCX 34 KB)

Supplementary material 2 (DOCX 29 KB)

Supplementary material 3 (DOCX 26 KB)

Supplementary material 4 (DOCX 29 KB)

Supplementary material 5 (DOCX 35 KB)

Supplementary material 6 (DOCX 13 KB)

Supplementary material 7 (DOCX 72 KB)
