# Supplementary material for: Thought–Action Fusion in Individuals with a History of Recurrent Depression and Suicidal Depression: Findings from a Community Sample
Source: Cognit Ther Res. 2018 Jun 4;42(6):782–93. doi: 10.1007/s10608-018-9924-7 (PMC6208973; doi:10.1007/s10608-018-9924-7)
Supplement: Supplementary file 1 — Supplementary material 1 (DOCX 34 KB) [file 10608_2018_9924_MOESM1_ESM.docx]

**Table S1**

*Baseline Characteristics of Sample*

|  |  | Groups |  |  |
| --- | --- | --- | --- | --- |
|  | Healthy Control | Depressed Non-Suicidal | Depressed Suicidal | *p*-value† |
| *n* (*%* of total) | 130 (36.01) | 134 (37.12) | 97 (26.87) | - |
| Female, *n* (*%*) | 92 (71.32) | 57 (42.54) | 71 (73.20) | <0.001 |
| Ethnicity, *n* (*%*) |  |  |  |  |
| White | 116 (89.23) | 84 (62.69) | 88 (90.72) |  |
| Asian | 14 (10.77) | 21 (15.67) | 7 (7.22) |  |
| Black | 0 (0) | 29 (21.64) | 2 (2.06) | <0.001 |
| Employment, *n* (*%*) |  |  |  |  |
| Employed | 125 (96.15) | 123 (91.79) | 83 (85.57) |  |
| Unemployed | 5 (3.85) | 11 (8.21) | 14 (14.43) | 0.017 |
| Age, *M* (*SD*) | 36.14 (12.80) | 35.58 (9.42) | 38.22 (11.43) | 0.196 |
| Education, *M* (*SD*) | 4.62 (1.27) | 4.24 (1.02) | 4.59 (0.98) | 0.010 |
| FFMQ, *M* (*SD*) |  |  |  |  |
| Total | 82.91 (11.62) | 75.02 (7.28) | 76.88 (10.66) | <0.001 |
| Non-Reacting | 15.94 (3.59) | 16.01 (2.50) | 15.82 (2.96) | 0.896 |
| Acting Awareness | 17.18 (3.27) | 15.34 (2.96) | 15.51 (3.54) | <0.001 |
| Observing | 14.55 (3.49) | 12.66 (3.20) | 13.65 (4.06) | <0.001 |
| Describing | 18.17 (3.48) | 15.99 (3.50) | 17.84 (3.33) | <0.001 |
| Non-Judging | 17.06 (3.68) | 15.02 (3.51) | 14.06 (3.61) | <0.001 |
| RRS, *M* (*SD*) |  |  |  |  |
| Total | 37.02 (9.32) | 50.34 (10.94) | 42.38 (11.87) | <0.001 |
| Reflection | 7.78 (2.68) | 11.28 (2.69) | 9.44 (3.12) | <0.001 |
| Brooding | 9.00 (2.55) | 11.26 (2.67) | 10.19 (3.22) | <0.001 |
| Depression | 20.25 (5.33) | 27.79 (6.97) | 22.75 (6.51) | <0.001 |
| ASIQ, *M* (*SD*) | 6.29 (9.66) | 21.51 (13.09) | 22.74 (19.87) | <0.001 |
| WBSI, *M* (*SD*) | 42.58 (10.35) | 44.51 (7.84) | 39.03 (11.85) | <0.001 |

Note: † P-values are based on χ^2^ tests for categorical variables and on ANOVAs for continuous variables with the exception of INQ, for which a t-test was use
