# Supplementary material for: Thought–Action Fusion in Individuals with a History of Recurrent Depression and Suicidal Depression: Findings from a Community Sample
Source: Cognit Ther Res. 2018 Jun 4;42(6):782–93. doi: 10.1007/s10608-018-9924-7 (PMC6208973; doi:10.1007/s10608-018-9924-7)
Supplement: Supplementary file 2 — Supplementary material 2 (DOCX 29 KB) [file 10608_2018_9924_MOESM2_ESM.docx]

**Table S2**

*TAF Item Ratings on whether Items are Extraneous (0) versus Self-Determined (100) and Negative (0) versus Positive (100) in Valence*

|  |  | Mean (SD) | |
| --- | --- | --- | --- |
| No | Item | Extraneous vs. Self-Determined | Negative vs. Positive |
| 1 | having fun on a holiday | 73.8 (18.9) | 81.5 (18.5) |
| 2 | donating to charity | 84.3 (19.9) | 75.1 (20.5) |
| 3 | eating healthily | 82.1 (17.8) | 78.1 (18.6) |
| 4 | lying to someone | 79.2 (23.3) | 25.9 (19.6) |
| 5 | cheating on my taxes | 79.7 (25.6) | 20.8 (19.7) |
| 6 | deliberately parking illegally in a disabled car parking space | 80.5 (27.1) | 23.4 (20.0) |
| 7 | winning the lottery | 16.4 (22.9) | 73.3 (25.2) |
| 8 | a stranger doing something kind for me | 27.8 (31.9) | 76.8 (23.7) |
| 9 | finding money on the street | 18.2 (21.6) | 62.6 (21.9) |
| 10 | becoming ill | 34.5 (20.7) | 20.7 (18.4) |
| 11 | house being burgled | 25.7 (23.4) | 16.7 (18.4) |
| 12 | being in a plane crash | 14.6 (22.2) | 13.1 (20.3) |
| 13 | killing myself | 70.0 (30.9) | 9.6 (16.3) |
| 14 | harming myself | 72.2 (27.8) | 12.9 (16.8) |
| 15 | harming myself with the intention to die | 72.3 (28.6) | 11.8 (17.5) |
