# Supplementary material for: Thought–Action Fusion in Individuals with a History of Recurrent Depression and Suicidal Depression: Findings from a Community Sample
Source: Cognit Ther Res. 2018 Jun 4;42(6):782–93. doi: 10.1007/s10608-018-9924-7 (PMC6208973; doi:10.1007/s10608-018-9924-7)
Supplement: Supplementary file 3 — Supplementary material 3 (DOCX 26 KB) [file 10608_2018_9924_MOESM3_ESM.docx]

**Table S3: Correlation Matrix between TAF-SR and Other Scales**^1)^

|  | TAF: Total | TAF: Un-controllable | TAF: Self-suicidal | TAF: Positive Controllable |
| --- | --- | --- | --- | --- |
| TAF: Total | 1 |  |  |  |
| TAF: Un-controllable | 0.97* | 1 |  |  |
| TAF: Self-suicidal | 0.68* | 0.53* | 1 |  |
| TAF: Positive Controllable | 0.23* | 0.06 | 0.11* | 1 |
| WBSI | 0.18* | 0.19* | 0.11* | -0.05 |
| SMQ | -0.19* | -0.20* | -0.20* | 0.12* |
| RRS | 0.62* | 0.64* | 0.45* | -0.07 |
| ASIQ | 0.51* | 0.50* | 0.45* | -0.08 |
| BRFL | -0.17* | -0.22* | 0.06 | -0.02 |
| FFMQ | -0.29* | -0.29* | -0.33* | 0.15* |

Note: Scales refer to Thought-Action Fusion (TAF), Southampton Mindfulness Questionnaire (SMQ), White Bear Suppression Inventory (WBSI), Ruminative Responses Scale (RRS), Five-Factor Mindfulness Questionnaire (FFMQ), Adult Suicidal Ideation Questionnaire (ASIQ), and Brief Reasons for Living Inventory (BRFL). * P<0.05.
