# Supplementary material for: Thought–Action Fusion in Individuals with a History of Recurrent Depression and Suicidal Depression: Findings from a Community Sample
Source: Cognit Ther Res. 2018 Jun 4;42(6):782–93. doi: 10.1007/s10608-018-9924-7 (PMC6208973; doi:10.1007/s10608-018-9924-7)
Supplement: Supplementary file 4 — Supplementary material 4 (DOCX 29 KB) [file 10608_2018_9924_MOESM4_ESM.docx]

**Table S4**

*Correlation Matrix between TAF 20-Item and RRS, FFMQ and WBSI Subscales*

|  | TAF: Total | TAF: Un-controllable | TAF: Self-suicidal | TAF: Positive Controllable |
| --- | --- | --- | --- | --- |
| RRS: Total | 0.62* | 0.64* | 0.45* | -0.07 |
| RRS: Ref† | 0.59* | 0.58* | 0.45* | 0.00 |
| RRS: Bro† | 0.43* | 0.45* | 0.34* | -0.09 |
| RRS: Dep† | 0.62* | 0.64* | 0.43* | -0.09 |
| FFMQ: Total | -0.29* | -0.29* | -0.33* | 0.15* |
| FFMQ: NR† | 0.00 | -0.01 | -0.08 | 0.14* |
| FFMQ: AA† | -0.37* | -0.34* | -0.35* | -0.04 |
| FFMQ: DS† | -0.40* | -0.42* | -0.32* | 0.14* |
| FFMQ: NJ† | 0.02 | 0.02 | -0.09 | 0.16* |
| FFMQ: OB† | 0.07 | 0.00 | 0.03 | 0.37* |
| WBSI: Total | 0.18* | 0.19* | 0.11* | -0.05 |
| WBSI: TS† | -0.05 | -0.06 | 0.00 | -0.03 |
| WBSI: UIT† | 0.38* | 0.40* | 0.31* | -0.14* |
| WBSI: SD† | -0.08 | -0.08 | -0.10 | 0.07 |

Note: † RRS subscale codes refer to reflection (Ref), Brooding (Bro), and Depression (Dep). FFMQ Subscales refer to Non-reacting (NR), Act with Awareness (AA), Describing (DS), Non-judging (NJ), and Observing (OB). WBSI subscale codes refer to thought suppression (TS), unwanted intrusive thoughts (UIT), and self-distraction. * P<0.05
