# Supplementary material for: Thought–Action Fusion in Individuals with a History of Recurrent Depression and Suicidal Depression: Findings from a Community Sample
Source: Cognit Ther Res. 2018 Jun 4;42(6):782–93. doi: 10.1007/s10608-018-9924-7 (PMC6208973; doi:10.1007/s10608-018-9924-7)
Supplement: Supplementary file 5 — Supplementary material 5 (DOCX 35 KB) [file 10608_2018_9924_MOESM5_ESM.docx]

**Table S5**

*Association between TAF-SR and Group*

| Sample |  | | Linear Regression Adjusted for Age and Gender | |
| --- | --- | --- | --- | --- |
|  | No. | Mean (SD) | Mean Difference [95% CI] | *P*-Value |
| **TAF: Total** |  |  |  |  |
| Healthy Controls | 130 | 36.49 (12.86) | [reference] | - |
| Depressed Non-Suicidal | 134 | 51.99 (15.65) | 15.50 [11.99, 19.02] | <0.001 |
| Depressed Suicidal | 97 | 38.48 (13.25) | 2.29 [-1.42, 6.00] | 0.159 |
| **TAF: Uncontrollable** |  |  |  |  |
| Healthy Controls | 130 | 20.69 (10.70) | [reference] | - |
| Depressed Non-Suicidal | 134 | 34.78 (12.63) | 13.87 [10.99, 16.75] | <0.001 |
| Depressed Suicidal | 97 | 21.24 (10.96) | 0.75 [-2.29, 3.80] | 0.576 |
| **TAF: Self-Suicidal** |  |  |  |  |
| Healthy Controls | 130 | 5.35 (3.12) | [reference] | - |
| Depressed Non-Suicidal | 134 | 7.77 (2.98) | 2.44 [1.66, 3.22] | <0.001 |
| Depressed Suicidal | 97 | 7.23 (3.45) | 1.99 [1.16, 2.81] | <0.001 |
| **TAF: Positive Controllable** |  |  |  |  |
| Healthy Controls | 130 | 10.45 (2.68) | [reference] | - |
| Depressed Non-Suicidal | 134 | 9.45 (2.11) | -0.81 [-1.45, -0.17] | 0.014 |
| Depressed Suicidal | 97 | 10.02 (2.93) | -0.45 [-1.13, 0.22] | 0.133 |

Note: Since the D-S group was similar to healthy controls in uncontrollable TAF and similar to the D-NS group for self-suicidal TAF, this could point towards a high specificity of TAF for self relevant, suicidal thoughts over more general, uncontrollable TAF in individuals with a history of suicidal depression. Therefore, we explored this specificity statistically by computing a difference score between mean self-suicidal and uncontrollable TAF (i.e., higher, positive scores indicate specificity towards self-suicidal TAF; lower, negative scores indicate specificity towards uncontrollable TAF). Results from these exploratory analyses (see our analysis code and results) demonstrated that the D-S group could be significantly differentiated from both other groups when looking at their specificity towards self-suicidal over uncontrollable TAF. This suggests a unique profile of lower uncontrollable and higher self-suicidal TAF in individuals with a history of suicidal depression.
