# Supplementary material for: Thought–Action Fusion in Individuals with a History of Recurrent Depression and Suicidal Depression: Findings from a Community Sample
Source: Cognit Ther Res. 2018 Jun 4;42(6):782–93. doi: 10.1007/s10608-018-9924-7 (PMC6208973; doi:10.1007/s10608-018-9924-7)
Supplement: Supplementary file 6 — Supplementary material 6 (DOCX 13 KB) [file 10608_2018_9924_MOESM6_ESM.docx]

**Table S6**

*Association of Group on TAF Change from Normal to Crisis State*

|  | Mean (SD) | | | | | |  |
| --- | --- | --- | --- | --- | --- | --- | --- |
|  | Depressed Non-Suicidal | | | Depressed Suicidal | | |  |
| TAF Scale | Normal | Crisis | Change‡ | Normal | Crisis | Change‡ | P-Value† |
| Total | 51.99 (15.65) | 53.57 (11.95) | 1.57 (13.49) | 38.48 (13.25) | 41.05 (11.96) | 2.57 (10.14) | 0.042 |
| Uncontrollable | 34.78 (12.63) | 36.70 (9.91) | 1.93 (9.87) | 21.24 (10.96) | 24.01 (10.89) | 2.77 (7.66) | 0.010 |
| Self-Suicidal | 7.77 (2.98) | 8.07 (2.69) | 0.30 (3.45) | 7.23 (3.45) | 9.60 (3.41) | 2.37 (3.39) | 0.028 |
| Positive Controllable | 9.45 (2.11) | 8.80 (1.90) | -0.65 (2.86) | 10.02 (2.93) | 7.44 (2.79) | -2.58 (3.43) | 0.012 |

Note: As done in Table S5, we again performed further exploratory analyses to see the change in TAF specificity towards self-suicidal when compared to uncontrollable content from normal to crisis states. Results demonstrated significant increases in self-suicidal TAF specificity in the D-S group when compared to the D-NS group (see our analysis code and results). This suggests that the specificity towards self-relevant, suicidal content in individuals with history of suicidal depression might further increase in states of crisis. † P-values are based on permutation-based linear regression analysis of the respective TAF crisis scale with predictors being group, respective normal state TAF, and their interaction. Thus, this can be interpreted as one group changing significantly more or less from normal to crisis state as is visible when looking at change columns. ‡ The change score describes the difference between respective TAF scores from normal to crisis states. * TAF: Self-Suicidal Specificity was computed for an additional, exploratory analysis.
