# Supplementary material for: Thought–Action Fusion in Individuals with a History of Recurrent Depression and Suicidal Depression: Findings from a Community Sample
Source: Cognit Ther Res. 2018 Jun 4;42(6):782–93. doi: 10.1007/s10608-018-9924-7 (PMC6208973; doi:10.1007/s10608-018-9924-7)
Supplement: Supplementary file 7 — Supplementary material 7 (DOCX 72 KB) [file 10608_2018_9924_MOESM7_ESM.docx]

# Figure 1


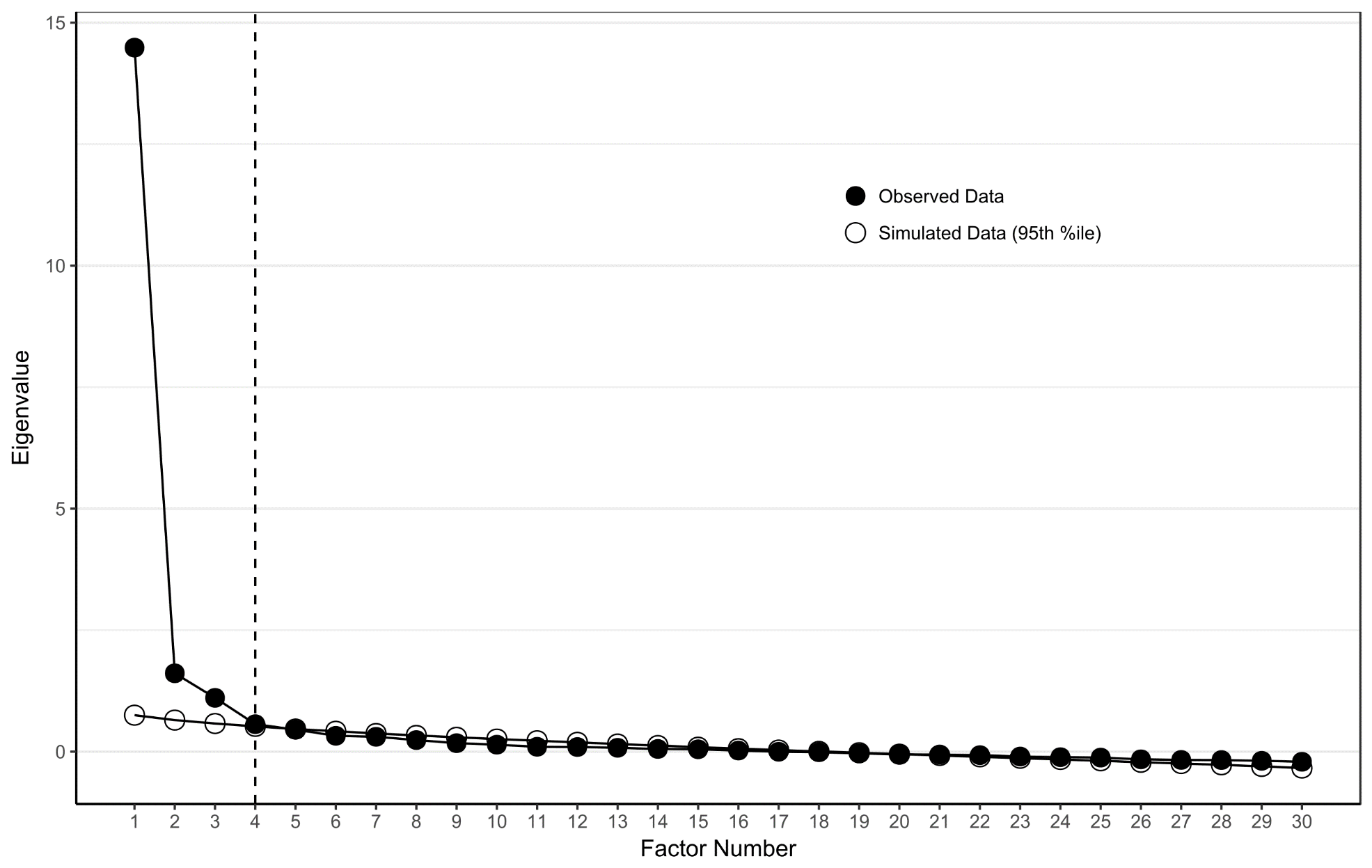


**Figure S1: Scree plot with results from principal axis factor analysis for the full sample (n=361)**
